# Targeting glutamine metabolism to modulate macrophage functions in the tumor microenvironment

**DOI:** 10.1007/s12672-026-04999-x

**Published:** 2026-04-24

**Authors:** Shuyan Wu, Yuwei Peng, Qian Wang, Zhaorong Li, Kewei Ge, Lihong Ye

**Affiliations:** 1 Department of Oncology, Liyang Hospital of Traditional Chinese Medicine, Liyang, 213300 Jiangsu China; 2 The First Clinical Medical College, Nanjing University of Chinese Medicine, Nanjing, China

**Keywords:** Glutamine metabolism, Tumor-associated macrophages, Tumor microenvironment, Cancer therapy

## Abstract

The increased glutamine demand of malignant cells imposes profound metabolic pressure on immune cells, particularly macrophages, forcing adaptive changes in their glutamine utilization pathways. This metabolic reprogramming involves three interconnected processes, namely, glutamine biosynthesis, catabolic breakdown, and cellular uptake, which are precisely regulated by specialized enzymes, metabolic intermediates, and membrane transport systems. As a pivotal area in modern oncology, glutamine metabolism serves as a master regulator of macrophage biology, simultaneously governing their bioenergetic requirements and immunological competence, thereby critically influencing tumor progression and metastatic potential. Our examination further reveals the intricate involvement of glutamine metabolic pathways in shaping the immunosuppressive tumor microenvironment and their dynamic interactions with host anti-tumor immune responses. These insights illuminate the therapeutic potential of targeting glutamine metabolism as an innovative strategy to improve cancer treatment paradigms.

## Introduction

The advent of single-cell RNA sequencing, spatial transcriptomics, and metabolomics has revolutionized our understanding of tumor biology, shifting research focus from cancer cells to the dynamic ecosystem of the tumor microenvironment (TME). This specialized niche comprises malignant cells and diverse non-neoplastic elements—immune cells, stromal components, and vascular networks—all embedded within a remodeled extracellular matrix [1]. Tumor cells subvert normal cellular functions, suppressing anti-tumor immunity while stimulating surrounding cells to support tumor progression, invasion, and metastatic dissemination through intricate cell–cell interactions and paracrine signaling [2].

Among TME constituents, immune cells [3], particularly myeloid lineage cells [4], play pivotal roles in cancer pathogenesis. As significant components of myeloid cells, macrophages exhibit remarkable functional plasticity in tumor settings [5]. With respect to tumor-associated macrophages (TAMs), these cells exhibit context-dependent behaviors that are capable of either restraining or promoting malignant progression. The pro-tumor activities of TAMs are multifaceted and include fostering immunosuppression, sustaining cancer cell proliferation, facilitating angiogenesis, and enabling metastatic spread [6, 7]. Notably, macrophages can express an array of inhibitory immune checkpoint molecules, including, but not limited to, PD-L1, PD-L2, B7-H4. They contribute to tumor progression through repressing T cell and NK cell activity, promoting an immunosuppressive TME [8–12]. Conversely, their anti-tumor potential manifests through direct tumor cell phagocytosis and killing, and indirect immune activation by antigen presentation [12, 13]. Whether TAMs exert anti-tumor or pro-tumor effects depends on their polarization status and subtypes. The functional polarization of TAMs is profoundly influenced by the unique metabolic landscape of the TME [14]. For instance, acidity and lactate tend to drive TAMs towards an anti-inflammatory and tumor supporting phenotype [15, 16]. This distinct physicochemical environment features altered glucose utilization, amino acid flux, lipid metabolism, energy production pathways, and a series of cytokines and chemokines. Recent studies revealed that metabolic reprogramming, such as CD40-mediated preferential fatty acid and glutamine utilization over glycolysis, can transform macrophages into anti-tumor phenotypes [17].

In the context of muscle regeneration, macrophage-derived glutamine can contribute to metabolic crosstalk and influence surrounding cells [18]. In the TME, glutamine becomes critically important through “glutamine addiction”—the increased dependence of cancer cells on this metabolite [19]. This metabolic competition creates glutamine scarcity in the TME, significantly impacting TAM biology. Glutamine deprivation impairs macrophage energetics by limiting α-ketoglutarate(α-KG) production for the TCA cycle while altering immune functions through multiple mechanisms [20]. In hepatocellular carcinoma models, glutamine-derived aspartate promotes HIF-1α translation, enhancing glycolytic flux and pro-inflammatory macrophage polarization, ultimately modulating their immunoregulatory functions [21]. Furthermore, glutamine modulates TAMs through the secretion of cytokines (TNF-α, IL-1, and IL-6) and mTORC1 signaling pathway regulation [22]. These findings collectively illustrate how glutamine orchestrates comprehensive changes in metabolic programming and immune functionality within macrophages through distinct pathways. This multifaceted regulation significantly impacts the broader TME, highlighting glutamine's central role in shaping tumor immunometabolism.

This review synthesizes the current understanding of how glutamine metabolism regulates TAM energetics and immune functions within the TME, while exploring the therapeutic potential of targeting these pathways for cancer treatment. By elucidating the metabolic control of macrophage polarization and activity, we aimed to identify novel strategies for disrupting tumor-promoting microenvironments.

## Glutamine metabolism of tumor cells in the TME

Research on tumor-related metabolism has revealed that numerous types of tumor cells in the TME exhibit a phenomenon termed “glutamine addiction” [23–26], a crucial feature of metabolic reprogramming in cancer. Among various malignancies, pancreatic cancer cells strongly rely on glutamine metabolism [27–29]. Specifically, tumor cells upregulate glutamine uptake and utilization to replenish TCA cycle intermediates, fulfilling their increased demands for energy production and nutrient metabolism [30]. In addition to serving as a source of α-KG to sustain the TCA cycle, glutamine-derived metabolites also facilitate the biosynthesis of amino acids, nucleotides, and fatty acids [31]. Consequently, depriving these cells of glutamine leads to growth arrest and cell death.

Enhanced glutamine metabolism is the primary driver of the addiction of tumor cells to glutamine within the TME. Glutamine metabolism involves the enzymatic conversion of glutamine into TCA cycle intermediates through multiple steps. First, glutamine is transported across the cell membrane via amino acid transporters such as SLC1A5 and SLC7A5 [32–34]. It is then hydrolyzed by glutaminase (GLS, an enzyme that helps break down glutamine to support cell energy and growth, and including GLS1 and GLS2) to glutamate, which can combine with cysteine and glycine to synthesize glutathione, scavenging reactive oxygen species (ROS) in the TME. Finally, glutamate is converted into α-KG by glutamate dehydrogenase (GDH) or transaminases. In addition to fuelling the TCA cycle with α-KG, glutamine metabolism supplies critical intermediates for the biosynthesis of macromolecules, including nucleotides, amino acids, and fatty acids. Glutamine-derived nitrogen contributes to de novo nucleotide synthesis (purines and pyrimidines) and supports NADPH production [30, 35]. Moreover, glutamine serves as a nitrogen donor for non-essential amino acids, promoting the synthesis of aspartate, alanine, and serine [36–39]. Clinical studies in breast cancer, colon adenocarcinoma, hepatocellular carcinoma, and prostate cancer have linked GLS overexpression to poor prognosis [40–43], underscoring the pivotal role of glutamine-derived nitrogen in tumor progression. Furthermore, glutamine provides carbon skeletons for fatty acid synthesis: α-KG is converted to citrate through a series of reactions, ultimately generating fatty acids. In conclusion, these mechanisms drive the increased dependency of cancer cells on glutamine to sustain their proliferative demands.

Emerging research has shown that glutamine addiction—a metabolic reprogramming phenomenon—is regulated by multiple oncogenes and tumor suppressor genes, including *c-MYC*, *KRAS*, *p53*, and *HIF* [31]. Substantial evidence has demonstrated that the proto-oncogene *c-MYC* upregulates the expression of amino acid transporters (SLC1A5, SLC7A6, and SLC7A5) involved in glutamine metabolism [44–46], thereby increasing glutamine uptake. In addition, *c-MYC* overexpression increases mitochondrial GLS expression, increasing glutamine catabolism [46, 47]. This metabolic shift activates the mTORC1 signaling pathway to promote tumor cell proliferation [48]. Similarly, the *KRAS* oncogene augments glutamine metabolism by upregulating SLC1A5 and SLC7A5 expression [49]. Furthermore, *KRAS* suppresses GDH while increasing glutamate–oxaloacetate transaminase (GOT) activity, facilitating ME1-mediated aspartate conversion to NADPH in the cytosol [50] to sustain the metabolic demands of tumors. Notably, the tumor suppressor *p53* modulates glutamine metabolism by inducing GLS2 expression while reducing intracellular ROS levels, thereby protecting genomic integrity [51]. Other tumor suppressors such as *LKB1* and *RB* impair glutamine uptake by suppressing SLC1A5 expression through E2F transcription factor 3 (E2F3) [52]. In summary, oncogenes and tumor suppressor genes orchestrate glutamine addiction through transporter regulation and metabolic enzyme modulation, revealing that glutaminolysis has therapeutic potential at the intersection of oncogenic signaling and metabolic adaptation.

## Glutamine and energy metabolism in macrophages

Increased glutamine uptake by tumor cells leads to decreased glutamine concentrations in the TME [53], resulting in altered macrophage states and functions. A key underlying mechanism is that glutamine deprivation impairs macrophage energy metabolism. As highly metabolically active cells, macrophages require sustained substrate availability to maintain their metabolic demands. Studies have demonstrated that macrophages rely on reprogrammed glucose, lipid, and amino acid metabolism for energy in the TME [54]. Even under aerobic conditions, tumor cells tend to utilize inefficient glycolysis for energy production—a metabolic hallmark known as the “Warburg effect” [55], which is also the primary metabolic pathway of M1-polarized macrophages [56, 57]. Macrophages exist in two polarized states: M1 macrophages exert anti-tumor effects by promoting inflammation, whereas M2 macrophages support tumor growth through immunosuppressive functions [58]. Metabolic profiling has emerged as a strategy to distinguish these phenotypes. Current evidence indicates that M2 macrophages predominantly depend on glutamine metabolism to fuel oxidative phosphorylation (OXPHOS, the process in cells that uses oxygen and nutrients to produce energy) and fatty acid oxidation (FAO) for their bioenergetic needs [56, 57].

Glutaminolysis, a central process in amino acid metabolism, critically regulates the energy supply of macrophages [14]. Mitochondrially derived α-KG from glutamine enters the TCA cycle to generate NADH for the electron transport chain (ETC), driving ATP production [58]. Moreover, α-KG serves as a substrate for de novo fatty acid synthesis in the TME, and FAO-derived ATP is a major energy source for M2 macrophages [59]. In addition, excessive glutamine consumption by tumor cells can impair mitochondrial fission in macrophages, reducing their energy output and phagocytic capacity [60]. Notably, both glutamine and lactate in the TME utilize IDH1 and ME1 to generate NADPH, supporting cellular energetics through interconnected pathways [35]. On the one hand, glutamine becomes the primary lactate source via the α-KG → TCA cycle → malate-pyruvate pathway → LDHA-mediated conversion under glucose deprivation [61], and lactate subsequently activates the mitochondrial ETC for ATP synthesis [62]. On the other hand, lactate enhances c-MYC activity [63], thereby increasing SLC1A5 and GLS1 expression to promote glutamine uptake and catabolism [64, 65]. Consequently, glutamine scarcity in the TME disrupts macrophage energy homeostasis, ultimately compromising their effector functions.

## Glutamine metabolism and macrophage immune functions

Glutamine serves as a critical energy source for macrophages. In addition to fueling metabolic demands, its depletion in the TME alters cytokine profiles and signaling pathways [66, 67], impairing macrophage immune functions. Studies have indicated that glutamine influences phagocytosis by regulating intracellular ATP levels [68]. Additionally, the functions of macrophages such as antigen presentation, immunosuppression and participation in tumor angiogenesis are also regulated by glutamine (Table 1).

**Table 1:** Glutamine metabolism-related genes and macrophage function

Gene	Glutamine metabolism process	Downstream genes and pathways	Macrophage function	Tumor phenotype	References
SLC7A8	Uptake	mTORC1/c-MYC, CD47	Phagocytosis	Proliferation	[82]
SLC1A5	Uptake	ROS, Thbs1, p38, Akt and SAPK/JNK	Immunosuppression	Proliferation, migration, invasion	[93, 117, 118]
SLC7A1	Upta#ke		Immunosuppression	Proliferation	[94]
GLS	Catabolism	MyD88/TRIF	Antigen presentation	Proliferation	[88]
		TNF,IL-10,NF-κB, STAT3	Immunosuppression	Proliferation, migration, invasion	[88, 90–92]
		NF-κB p65, IQGAP1	Tumor angiogenesis	migration	[102]
GS	Anabsolism	CD69	Immunosuppression	Proliferation, migration, invasion	[109]
		CD31, REDD1, HIF-1α and mTOR	Tumor angiogenesis	Proliferation, migration, invasion	[109, 110]

### Phagocytosis

TME-resident macrophages require continuous glutamine uptake to sustain their hypermetabolic state. Research has demonstrated that the glutamine concentration directly correlates with phagocytic capacity of macrophages in Nile tilapia [69]. In vitro, murine peritoneal macrophages exhibit increased phagocytosis with increasing glutamine concentrations, plateauing near physiological plasma levels [70]. In vivo, studies in tumor-bearing rats revealed that glutamine infusion restores the diminished phagocytic ability of alveolar macrophages against sheep red blood cells (SRBCs) [71]. These findings confirming that glutamine deprivation compromises phagocytic function [70].

Phagocytosis is an energy-intensive process that is dependent on intact cytoskeletal dynamics; structural disruption impairs engulfment [72]. Fletcher et al. [73] found that ATP depletion alters cytoskeletal organization, whereas glutamine preserves ATP levels, safeguarding phagocytic function [68]. In addition to cytoskeletal integrity, effective phagocytosis depends on surface receptor-mediated recognition of tumor and apoptotic cells within the TME. Dini et al. [74] observed a positive correlation between surface receptor expression levels and phagocytic efficiency in rat hepatic Kupffer cells. Spittler et al. [75] reported that glutamine restriction downregulates key phagocytic receptors, particularly high-affinity IgG and CR3. And this diminished the capacity of peripheral blood mononuclear macrophages for IgG-mediated phagocytosis. Research has indicated that glutamine-derived nitrogen protects against CR3 expression in microglia, maintaining their phagocytic activity [76].

During early tumor infiltration, macrophages actively engulf tumor cells; however, this phagocytic capacity becomes progressively suppressed with tumor progression [77]. CD47, a ubiquitously expressed cell surface immunoglobulin, is expressed at significantly higher levels on tumor cells than on normal cells [78, 79]. In-depth studies revealed that tumor cell surface CD47 engages macrophage signal regulatory protein α (SIRPα), delivering an immunoregulatory “don’t eat me” signal that facilitates immune evasion by inhibiting phagocytosis [80]. Consequently, blockade of the CD47-SIRPα axis enhances macrophage-mediated tumor cell clearance and exerts anti-tumor effects [78, 79, 81]. Notably, in osteosarcoma models, the modulation of L-type amino acid transporter 2 (LAT2)-dependent glutamine uptake activates both mTORC1 signaling and c-MYC-driven CD47 transcription, ultimately suppressing macrophage phagocytosis and promoting immune escape [82]. These findings indicate a context-dependent duality: while glutamine normally potentiates macrophage phagocytic function, it paradoxically activates the anti-phagocytic pathway and promotes immune suppression within the TME. Therefore, targeting this pathway could restore macrophage tumoricidal activity and inhibit malignant progression.

### Antigen presentation

As professional antigen-presenting cells (APCs), macrophages express abundant MHC class II (MHC-II) molecules. Through TCR-mediated engagement, they present antigens to CD4^+^ helper T cells, triggering T cell activation and cytokine production to initiate adaptive immune responses [83]. The immunomodulatory effects of macrophages exhibit context-dependent outcomes influenced by tumor type, grade, and TME status [84]. For instance, in bladder cancer models, macrophage-mediated antigen presentation to CD4^+^ T cells primes CD8^+^ T cell cytotoxicity [85]. Analogous findings were reported by Chen et al. in mesenchymal-like glioblastoma, where microglia function as APCs to activate CD4^+^ T cells while maintaining phagocytic activity [86]. Conversely, HLA-DR^+^ monocyte-derived macrophages may suppress CD4^+^ T cell responses, highlighting their functional plasticity [87].

Glutamine dependence governs macrophage APC function. In vitro studies have demonstrated that glutamine restriction impairs antigen presentation, inhibiting host immunity [75]. Paradoxically, pharmacologic inhibition of glutaminolysis (e.g., via the glutaminase inhibitor JHU083) enhances APC capacity by inducing immunogenic cell death (ICD), a type of cell death that triggers the immune system to attack cancer cells [88]. In the B16-OVA and MC38-OVA models, JHU083-treated macrophages upregulated MHC-II expression without stimulating CD8^+^ T cell proliferation, whereas pretreatment of tumor cells before co-culture significantly enhanced cytotoxic T cell responses. These findings suggest that optimal antigen presentation requires coordinated danger signals from dying tumor cells and metabolic reprogramming of macrophages. Furthermore, this process critically depends on intact MyD88/TRIF signaling and the lysosomal function of macrophages [88]. Collectively, these findings establish glutamine metabolism as a key regulator of macrophage-mediated T cell activation, offering potential therapeutic strategies to enhance anti-tumor immunity through metabolic modulation.

### Immunosuppression

Recent studies have identified excessive glutamine consumption as a metabolic hallmark of clear cell renal carcinoma, leading to impaired glutamine utilization by TAMs in the TME. Under glutamine-deprived conditions, TAMs stimulate HIF-1α-mediated IL-23 production, which subsequently induces regulatory T cells to secrete IL-10 and TGF-β, ultimately suppressing CD8^+^ T cell-mediated tumor killing [89]. Further research [56, 67] has demonstrated that glutamine modulates macrophage polarization and immunosuppressive function by remodeling cytokine networks, chemokine profiles, metabolic enzyme activity, and associated signaling pathways in the TME.

In vitro studies using BMDMs revealed that α-KG, a glutamine catabolite, drives JMJD3-dependent epigenetic reprogramming to promote M2 macrophage polarization and immunosuppression [90]. Specifically, α-KG facilitates JMJD3-mediated H3K27me3 demethylation, activating the transcription of immunosuppressive genes (*ARG1*, *YM1*, *RETNLA*, *MRC1*) to establish an immunosuppressive TME [90]. Complementary work by Lee et al. [91] showed that modulating the α-KG/succinate ratio regulates the JMJD3-IRF4 axis, with α-KG supplementation promoting M2 polarization. This establishes α-KG as a critical metabolic checkpoint in glutaminolysis. The immunosuppressive effects of glutamine inhibit M1 polarization through α-KG–dependent mechanisms. Glutamine-derived α-KG activates prolyl hydroxylases (PHDs), which suppress HIF-1α and IKKβ/NF-κB signaling, thereby limiting M1 macrophage development [90]. This is in agreement with other papers showing that reducing HIF-1α signaling limits M1 macrophage polarization [13]. Furthermore, pharmacologial inhibition of GLS1, which depletes glutamine, is characterized by reduced M2 gene expression and elevated expression of pro-inflammatory genes (*IL1B*, *IL6*, *IL12B*, and *TNF*) [90]. These findings support the targeting of glutamine metabolism to repolarize TAMs toward tumor-suppressive M1 phenotypes during anti-cancer therapy [92]. Consistent with these findings, Oh et al. [88] reported that JHU083 enhances NF-κB signaling and TNF secretion while reducing IL-10 production and STAT3 phosphorylation in LPS-stimulated BMDMs, suggesting that glutamine blockade promotes pro-inflammatory responses through NF-κB activation and STAT3 suppression [88, 90].

Additional regulation occurs through the glutamine transporters SLC1A5 and SLC7A11 [93, 94]. Li et al. [93] reported that blockade of SLC1A5 depletes glutathione, increases ROS, and activates Thbs1-mediated p38/Akt/JNK signaling in oral keratinocytes, ultimately driving M1-like TAM polarization via exosome-mediated crosstalk. These findings suggest that glutamine metabolism orchestrates an immunosuppressive TME by (1) epigenetically reprogramming macrophages toward M2 polarization via α-KG/JMJD3; (2) inhibiting M1 polarization through NF-κB suppression; (3) supporting immunosuppressive signaling via STAT3 activation; and (4) glutamine transporters such as SLC1A5, which can also contribute to immunosuppression. Taken together, these results highlight glutaminolysis as a therapeutic target to reverse TAM-mediated immunosuppression and restore anti-tumor immunity.

### Angiogenesis

M2-polarized macrophages serve as pivotal regulators of angiogenesis in the TME, functioning as the primary source of pro-angiogenic factors (VEGF, EGF, PDGF, TGF-α/β, Ang-1/2), extracellular matrix-degrading enzymes (MMPs) [59], and inflammatory cytokines (TNF-α, IL-1β, IL-8, COX-2). These mediators collectively promote pathological neovascularization, which sustains tumor growth and facilitates metastatic dissemination [95–98]. In turn, the blood vessel proximity can also influence the cancer cell metabolism, and potentially, further altering the TME and TAMs phenotype [99–101]. Moreover, glutamine metabolism drives the M2-like polarization of TAMs, suggesting its potential role in facilitating macrophage-mediated angiogenesis. In support of this hypothesis, studies in HER2-positive gastric cancer have demonstrated that CDC42 promotes M2 polarization and angiogenic programming through GLS1-dependent activation of the NF-κB p65 and IQGAP1 signaling pathways [102].

Glutamine synthetase (GS), the sole enzyme responsible for de novo glutamine biosynthesis from glutamate and ammonia, is critical for promoting tumor cell proliferation and metastatic progression [103, 104]. Emerging evidence has demonstrated that GS is frequently overexpressed across multiple cancer types, including hepatocellular carcinoma [105], glioma [106], ovarian cancer [107], and sarcoma [108]. It actively contributes to tumorigenesis and disease progression. In addition to its effects on cancer cells, GS significantly influences the TME through regulating the activity of fibroblasts, macrophages, and T lymphocytes. Notably, elevated GS expression and activity drive the expansion of M2-polarized macrophages, which play dual roles in (1) establishing an immunosuppressive microenvironment; and (2) facilitating tumor angiogenesis [109]. Palmieri et al. [109] provided evidence through in vitro studies showing that GS activation promotes IL-10–mediated M2 polarization; GS enhances immunosuppression by downregulating CD69 expression, inhibiting CD4^+^ and CD8^+^ T cell proliferation, and impairing CD8^+^ T cell migration. These findings were further corroborated by observations of increased M2 macrophage infiltration and elevated CD31^+^ microvessel density in GS-overexpressing tumors [109]. Furthermore, in vivo studies in melanoma models revealed that pharmacological GS inhibition can repolarize TAMs to the M1-like phenotype, attenuate immunosuppression, and disrupt tumor angiogenesis by downregulating of REDD1 expression and activating the HIF-1α/mTOR signaling pathway [110]. Thus, targeting glutamine anabolism modulates macrophage polarization and suppresses tumor angiogenesis, suggesting a novel therapeutic strategy focused on GS-mediated anti-angiogenesis effects.

SLC7A8, SLC1A5, and SLC7A11 facilitate the uptake of glutamine. Among these amino acid transporters, SLC7A8 can activate the mTORC1 signaling pathway and CD47 transcription, thereby inhibiting macrophage phagocytosis. SLC1A5 downregulates ROS by maintaining glutathione levels, subsequently inhibiting the p38/Akt/JNK signaling pathway and promoting M2 macrophage polarization, resulting in immunosuppression. Glutamine is then hydrolyzed by GLS1/GLS2 to glutamate. GLS1 can drive M2 polarization by suppressing TNF and NF-κB signaling and activating IL-10 and STAT3 phosphorylation, ultimately leading to immunosuppression. GLS1 can also promote macrophage polarization toward the M2 phenotype and help macrophages participate in tumor angiogenesis via NF-κB p65 and IQGAP1 activation. Finally, glutamate is converted into α-KG by GDH. α-KG drives M2 macrophage polarization through JMJD3-mediated and PHD-mediated pathways to facilitate immunosuppression. Furthermore, glutamine can be synthesized de novo by GS. GS can promote both immunosuppression and tumor angiogenesis through activating M2 macrophage polarization.

## Targeting glutamine metabolism in TAMs

Glutamine metabolic reprogramming represents a hallmark feature of the TME [111]. For instance, glutamine can activate mTORC1 through AMPK-dependent and α-KG–dependent pathways to drive tumor progression, making these pathways viable therapeutic targets [48]. In addition to tumor cells, glutamine sustains non-malignant TME components (e.g., immune cells and stromal cells) by fueling their energy demands and modulating immune responses [112]. Although tumor cells dominate glutamine consumption, their intrinsic heterogeneity [31] and propensity for therapy resistance [113, 114] have shifted their research focus toward targeting non-cancerous TME elements and associated pathways.

**Fig. 1:**
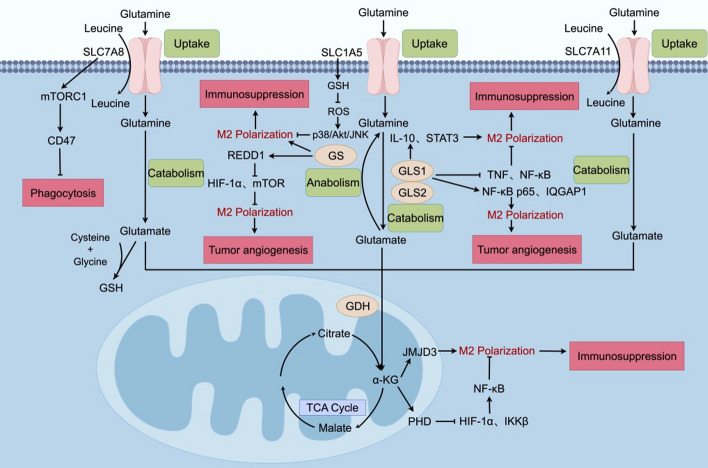
Glutamine metabolism related to tumor-associated macrophages

Emerging evidence has established glutamine and its metabolites as critical regulators of macrophage energy metabolism and immune function (Fig. 1). This understanding has spurred the development of molecularly targeted therapies against key enzymes and products in glutamine metabolic pathways as a novel strategy to combat tumor growth and metastasis. Among these approaches, targeting amino acid transporters such as LAT2 and SLC1A5, which mediate glutamine uptake has shown promise in modulating macrophage function for anti-tumor therapy. Pharmacological inhibition of LAT2 downregulates CD47 expression, enhances macrophage infiltration and phagocytic activity, and increases tumor cell sensitivity to chemotherapy, thereby improving therapeutic outcomes [82]. In hepatocellular carcinoma, elevated SLC1A5 expression is correlated with increased infiltration of immunosuppressive macrophages and is clinically associated with poor prognosis and treatment resistance, suggesting that SLC1A5 inhibitors may exert anti-tumor effects by disrupting M2 macrophage function [115, 116].

Significant research efforts have focused on manipulating glutamine catabolism to influence macrophage function through the use of GLS inhibitors and glutamine analogs. GLS1 inhibitors such as BPTES, 968, and CB-839 promote M1-like macrophage polarization while inhibiting tumor angiogenesis [102]. Preclinical studies have demonstrated that BPTES-mediated glutamine metabolic blockade reduces immunosuppressive M2-like and CX3CR1^+^ macrophage infiltration while increasing the infiltration of cytotoxic T lymphocytes and M1-like macrophages, effectively remodeling the immunosuppressive TME in cholangiocarcinoma. This approach has synergistic effects when combined with immune checkpoint inhibitors [117]. Similarly, compound 968 reprograms the TME by shifting macrophage polarization from the M2-like phenotype to the M1-like phenotype, leading to increased CTL recruitment and activation [118]. CB-839, the only GLS1 inhibitor in current clinical trials [119–121], shares this mechanism of action through the inhibition of M2-like polarization [122]. Glutamine analogs, which function primarily as antagonists of glutamine catabolism [123], induce ICD to enhance macrophage antigen presentation while increasing M1-like macrophage populations [88]. These compounds reverse immunosuppressive conditions in the TME and restore immune cell-mediated tumor killing [88, 90–92]. Early glutamine analogs such as DON show limited clinical potential due to their poor pharmacological properties and significant gastrointestinal toxicity; however, next-generation prodrugs such as DRP-104 demonstrate improved safety profiles while maintaining therapeutic efficacy [124]. DRP-104 promotes M1-like macrophage conversion and shows enhanced anti-tumor activity when combined with checkpoint inhibitors [125]. The DON prodrug JHU083 exhibits particular promise in genitourinary cancers, where its anti-tumor effects extend beyond T cell dependence to include reprogramming of TAMs toward a pro-inflammatory phenotype, enhanced phagocytic capacity, and inhibition of tumor angiogenesis [126]. In conclusion, these inhibitors and analogs vary in their mechanisms of action, selectivity, efficacy across different tumor types and potential side effects. CB-839 selectively inhibits GLS1 with high specificity and demonstrates mild side effects, such as reduced hematological toxicity. DRP-104 and JHU083 both competitively inhibit multiple glutamine-dependent enzymes in a non-selective manner and have more pronounced side effects compared to CB-839 [125, 127]. Regarding efficacy against different types of tumors, CB-839 is highly dependent on the genetic background of the tumor and has shown better efficacy in tumors caused by mutations such as KEAP1 [128]. However, due to its selective mechanism, tumors may develop resistance by activating alternative metabolic pathways [28]. In contrast, DRP-104 and JHU-083 are expected to exhibit potential efficacy against a variety of solid tumors owing to their broad-spectrum and potent inhibitory effects. It should be noted that JHU-083 has not yet entered clinical trials, and its clinical translation and ultimate human data remain to be validated.

Besides, glutamine synthetase inhibitors, including methionine sulfoximine (MSO) and glufosinate drive M1-like macrophage polarization, increase cytotoxic T lymphocyte accumulation, alleviate immunosuppression, and suppress tumor angiogenesis. Preliminary in vitro and in vivo studies have demonstrated their potent anti-tumor effects [109, 110]. In conclusion, these glutamine metabolism-targeting agents represent a clinically promising therapeutic approach that modulates macrophage immune functions and reshapes the TME to inhibit tumor initiation and progression. However, further research on the multifaceted mechanisms of these molecular-targeted drugs is warranted for cancer treatment applications.

## Discussion

This review comprehensively examines the phenomenon of glutamine addiction within the TME, focusing on the critical metabolites and enzymes involved in glutamine synthesis, uptake, and catabolism. These metabolic components significantly influence macrophage energy metabolism and immune functions, including phagocytic capacity, antigen presentation, immunosuppressive activity, and participation in tumor-associated angiogenesis. Furthermore, glutamine metabolism profoundly affects other TME components such as T lymphocytes, ultimately regulating tumor cell proliferation, survival, and metastatic potential. Targeted interventions against key metabolic nodes in glutamine pathways can effectively reprogram macrophage polarization states and functional phenotypes, suggesting promising strategies to inhibit tumor progression and metastasis.

The complex network of glutamine metabolism involves numerous molecular targets and signaling pathways. Tumor cells exhibit markedly increased glutamine demand, particularly within the TME, which restricts glutamine availability for non-malignant cells and compromises their metabolic and functional integrity. In addition to macrophages, glutamine metabolism regulates various immune populations, including T cells, neutrophils, cancer-associated fibroblasts (CAFs), and dendritic cells (DCs). For instance, combined administration of the GLS1 inhibitor CB-839 with 5-FU in IK3CA-mutant colorectal cancer models results in the recruitment of tumoricidal neutrophils that form extracellular traps to induce apoptotic cell death [129]. Among immune cells, T lymphocytes have been most extensively studied in the context of glutamine metabolism. Glutamine supports T cell proliferation and cytokine production, enhancing anti-tumor immunity [130]. The SLC1A5 inhibitor V-9302 has selective effects on triple-negative breast cancer cells while preserving CD8^+^ T cell glutamine uptake, ultimately improving T cell function through glutathione synthesis and exerting anti-tumor effects [42]. Moreover, other glutamine transporters like SLC7A5 and SLC25A22 also showed promise in enhancing CD8 + T cell function and potentiating the efficacy of anti-PD-1 therapy [131, 132]. A preclinical study in KEAP1-mutant lung cancer demonstrated that DRP-104 reverses T cell exhaustion, reduces Tregs, and enhances the function of CD4^+^ and CD8^+^ T cells, ultimately improving the response to anti-PD-1 therapy [128]. Compared with monotherapy, JHU083 significantly improves survival across multiple tumor models and shows superior efficacy when combined with PD-1 blockade, probably through increased CD8^+^ T cell infiltration [133]. This finding is consistent with the research results in EGFR gene-driven lung cancer [134]. However, contrasting results in Lkb1-deficient models, where CB-839 plus anti-PD1 suppresses CD8^+^ T cell activation [135], highlight the need for further research on glutamine-targeting agents. In addition, CB-839 showed no significant efficacy either alone or with other targeted drugs in a preclinical study on chronic lymphocytic leukemia [136]. These controversial data point to a question: whether targeting glutamine metabolism is beneficial or not for tumor growth? Most evidence indicates that targeting glutamine metabolism suppresses tumor growth and represents a clinically promising therapeutic strategy. However, certain studies report limited efficacy or even tumor-promoting effects. This discrepancy may be attributed to the metabolic flexibility of both tumor cells and immune cells within the TME, which can activate alternative pathways to evade glutamine addiction [28, 137]. The current developmental landscape of glutamine metabolism-modulating therapeutics reveals that several drugs like DRP-104 and CB839 are now in early-phase clinical trials (phase I/II). But most candidate drugs affecting the TME glutamine pathways remain confined to preclinical studies. This prevailing circumstance underscores the extended timeframe required for translational advancement of these experimental agents into clinically available anti-cancer medications.

While this review primarily explores how glutamine metabolic reprogramming affects macrophage immunobiology and how targeting these pathways can remodel the TME to combat cancer, we acknowledge certain limitations. We can’t detail the effects of glutamine on other TME components, although a comprehensive understanding of these interactions is essential for developing effective therapeutic strategies. The most promising metabolic targets involve key pathways governing glutamine metabolism in macrophages, T cells, neutrophils, CAFs, and even myeloid-derived suppressor cells (MDSCs). Research in the 4T1 triple-negative breast cancer model has found that targeting glutamine metabolism inhibits the recruitment of MDSCs and increases inflammatory TAMs. This further enhanced anti-tumor immunity and inhibited tumor growth and metastasis [88]. Furthermore, another study showed that glutamine synthesis in CAFs drives the polarization of pro-tumorigenic TAMs, fostering an immunosuppressive microenvironment that supports tumor growth. It reveals a novel metabolic symbiosis in which tumor cells exploit CAF-mediated glutamine metabolism to manipulate TAM polarization, highlighting a potential therapy for immunotherapy [138]. Ultimately, macrophage and T lymphocyte metabolism represent the most studied areas, with emerging evidence showing functional interdependence between these populations in the TME [89, 109]. Recent clinical experience has demonstrated that single-agent therapies targeting macrophage or T cell pathways (e.g., CSF-1R or PD-1 antibodies) often yield modest outcomes [139, 140]. This underscores the need for innovative combination approaches that simultaneo usly modulate multiple metabolic pathways in macrophages and T cells—an emerging paradigm. Future research should focus on rationally designed interventions that either coordinate metabolic targeting across immune cell populations or combine metabolic modulation with established immunotherapies. Such integrated strategies hold significant promise for overcoming current limitations in cancer treatment.

## Data Availability

No datasets were generated or analysed during the current study.
